# Matrix Metalloproteinase-9 Is a Predictive Factor for Systematic Hypertension and Heart Dysfunction in Patients with Obstructive Sleep Apnea Syndrome

**DOI:** 10.1155/2018/1569701

**Published:** 2018-03-06

**Authors:** Shuhui Wang, Shisheng Li, Bin Wang, Jiajia Liu, Qinglai Tang

**Affiliations:** Department of Otolaryngology, Head and Neck Surgery, The Second Xiangya Hospital, Central South University, Changsha, Hunan 410011, China

## Abstract

Patients with obstructive sleep apnea syndrome (OSAS) showed higher prevalence in cardiovascular diseases due to aberrant hypoxia and oxidative stress. However, not all OSAS patients end up with cardiovascular disorders, and identification of novel biomarker will be invaluable for differentiating patients at risk. Here we tested the serum matrix metalloproteinase-9 (MMP-9) levels in 47 untreated OSAS patients and found that the MMP-9 level was positively correlated with severity of OSAS, which was consistent with hypoxia degree and duration. Besides, the MMP-9 level was higher in patients complicated with systematic hypertension (*P* < 0.001). Furthermore, we selected those OSAS patients without any cardiovascular dysfunction (*n* = 35) and followed up for up to five years. By the end of follow-up, 12 patients had hypertension onset and 3 patients had left ventricular hypertrophy. By analyzing the clinical outcomes with MMP-9 expression, we demonstrated that high serum MMP-9 in OSAS patients was a risk factor for occurrence of cardiovascular diseases. In addition, we cultured the vascular endothelial cells (VEC) from rat aorta in hypoxia condition to investigate whether MMP-9 was elevated due to hypoxia in OSAS patients. Cellular results revealed that the expression, secretion, and activity of MMP-9 were all upregulated by hypoxia and can cleave the beta2-adrenergic receptor (*β*2AR) on VEC surface. Our results not only determined MMP-9 as a risk factor for cardiovascular diseases in OSAS patients, but also showed the possible involvement of hypoxia-MMP-9-*β*2AR signaling axis.

## 1. Introduction

Apnea is defined as the absence of inspiratory airflow for at least 10 seconds, which can be classified as obstructive or central [1]. Normal sleep is important for many aspects such as relaxing the physiological stress and protecting central nervous system and cardiovascular system [2]. Patients with OSAS suffered from disrupted sleep and dysregulated physical and psychological feelings. For example, the unfavorable consequences of OSAS include abnormal arterial blood gas components and decreased parasympathetic and increased sympathetic activity, all of which are harmful for cardiovascular system [3]. In fact, 30–83% of hypertension patients also suffered with OSAS [4]. Instead of just correlation between hypertension and OSAS, recent studies demonstrated a causal relationship between OSAS and hypertension. Since large amounts of adults are with OSAS (34% of men and 17% of women) [1], treating OSAS may make pronounced contributions to control the cardiovascular diseases incidence. However, not all OSAS patients end up with cardiovascular dysregulation; therefore, the first thing is to figure out risk factors of OSAS patients. It has been revealed that the intermit hypoxia caused by OSAS will up-regulate the serum inflammatory mediators [5]. Despite the importance of those serum molecular attracting more and more attention, which cytokine or which combination is helpful for clinical prediction remains unclear.

A recent study reported a statistical correlation between higher serum matrix metalloproteinase-9 (MMP-9) level and the progression of blood pressure in healthy individuals [6]. MMP-9 is a kind of enzyme belonging to the zinc-metalloproteinases family and functions in degrading the extracellular matrix [7]. The involvement of MMP-9 in cardiovascular diseases has been well-acknowledged [8–10]. Interestingly, an elevated MMP-9 secretion was reported to be induced by hypoxia conditions [11, 12], which triggered us to investigate whether and how serum MMP-9 in OSAS patients affects the risk of systematic hypertension and heart dysfunction.

Briefly, we enrolled 47 untreated OSAS patients and analyzed their clinicopathological characteristics. By testing the serum level of MMP-9 in these patients, we can tell its statistical correlation with OSAS severity. Similarly, whether patients complicated with hypertension showed higher MMP-9 levels was verified. We next followed up the cases without cardiovascular dysfunction to explore the effect of MMP-9 on the occurrence of hypertension and/or left ventricular hypertrophy in OSAS patients. Finally, we performed cellular and molecular experiments to investigate the possible functional mechanisms of MMP-9 in promoting cardiovascular diseases of OSAS patients.

## 2. Patients and Methods

### 2.1. Patients and Criteria

A total of 47 OSAS patients (11 females, 36 males, mean age 54.3 ± 7.2 years) were included in the study. All patients underwent standard hospital examinations, including physical and cardiological examination, laboratory examination, and standard nocturnal polysomnographic study. Diagnosis was based on cardiorespiratory sleep study (continuous cessation of airflow for ≥10 seconds). All patients were free from known metabolic disorders, respiratory infection, and other respiratory disorders at the time of diagnosis.

Severity of OSAS was classified based on the apnea hypopnea index (AHI) [13]: mild (5 ≤ AHI < 15), moderate (15 ≤ AHI < 30), and severe (30 ≤ AHI). The Epworth Sleepiness Scale (ESS) and oxygen desaturation index (ODI) were also scored for all the patients. Other retrieved clinicopathological parameters included body mass index (BMI) and neck and waist circumferences. Blood oxygen saturation (SpO_2_) parameters during sleep were monitored regarding lowest SpO_2_, mean SpO_2_, and minutes of SpO_2_ less than 90%. Blood samples were withdrawn in the morning around 8:00 a.m. to test the serum MMP-9 concentration. Moreover, based on the existence of hypertension or not at the time of diagnosis, we classified the 47 cases into two subgroups: patients with hypertension (*n* = 12) and patients without hypertension (*n* = 35). Patients in the latter group were followed up for up to five years to monitor occurrence of cardiovascular diseases. Written informed consent was obtained from all patients. This study was approved by the Ethics Committee of Second Xiangya Hospital.

### 2.2. Cell Culture and Enzyme-Linked Immunosorbent Assay (ELISA)

Rat aorta VEC cells were purchased from Creative Bioarray (Cat. No. CSC-C8696W, Creative Bioarray, Shirley, NY, USA). Cells were cultured in DMEM supplemented with 10% fetal bovine serum (FBS) at 37°C in a humidified 5% CO_2_ atmosphere. The cells were exposed to hypoxia in a hypoxic incubator (Heraeus HERAcell 240i; Thermo Scientific, USA) filled with 3% oxygen, 5% CO_2_, and 92% nitrogen at 37°C for indicated time periods. Normoxia cells were cultured parallelly in a normal condition (37°C with 5% CO_2_). The ELISA kit for MMP-9 (ELISA, R&D Systems Inc., Minneapolis, MN, USA) was used to quantify MMP-9 levels in the serum of OSAS patients' and conditional cultured medium of rat aorta VEC cells.

### 2.3. Reverse Transcription Quantitative Real-Time Polymerase Chain Reaction (RT-qPCR)

Total RNA was extracted from the cells using Trizol reagent (Thermo Fisher Scientific, Pittsburgh, PA, USA) and reversely transcribed by M-MLV reverse transcriptase (USB Corporation, USA). Quantitative real-time PCR was performed with followed cycling (95°C for 8 min, 40 cycles for 30 s at 95°C, 1 min at 55°C, and 30 s at 72°C) using ΔΔCt method on Mx-Pro Mx3005P v4.00 software. GAPDH was used for normalization. The PCR primers were as follows:

  MMP-9: 5′-ACC CTT GTG CTC TTC CCT-3′, and 5′-GGT TCG CAT GGC CTT CAG-3′;
  GAPDH: 5′-GAT TTG GCC GTA TCG GAC -3′, and 5′- GAA GAC GCC AGT AGA CTC -3′.

### 2.4. Gelatin Zymography

MMP-9 enzyme activity was measured using gelatin-containing gels as described by Kleiner and Stetler-Stevenson [14]. Briefly, samples with normalized protein concentration (50 *μ*g) were mixed with sample buffer (0.4 M Tris– HCl pH 6.8, 10% SDS, 20% glycerol and 0.1% bromphenol blue) and were separated by SDS-PAGE containing 0.1 mg/mL gelatin at 4°C. After electrophoresis, SDS was removed from the gel by washing twice with renaturing buffer (2.5% Triton X-100) at room temperature for a total 1 h. The gels were then incubated in the zymography buffer (50 mM Tris-HCl, pH 7.4, and 15 mM CaCl_2_) at 37°C overnight for maximum sensitivity. The Coomassie Blue R-250 staining and destaining were then carried out. Standards for the active recombinant MMP-9 protein were included on the gels as a positive control. Finally, the gelatinolytic activity was semiquantified by densitometric analysis using Image J software.

### 2.5. Western Blot

Cell pellets were resuspended with hypotonic lysis buffer (10 mM HEPES, pH 7.4, 2 mM EDTA, 10 mM MgCl_2_, 5 units/mL DNase, protease and phosphatase inhibitors) at 4°C for 30 min incubation and then centrifuged at 10,000*g* for 10 minutes to remove cell fragments; the supernatant was collected and subjected to 37,500*g* centrifugation for another 30 minutes to collect cell membrane in the precipitate. The membranes were further homogenized with membrane extraction buffer (20 mM HEPES, pH 7.4, 500 mM NaCl, 10 mM MgCl_2_, 5 units/mL DNase, 8% Glycerol, 1.0% DDM, protease and phosphatase inhibitors). Protein concentration was then quantified and about 25 *μ*g of proteins was resolved by 10% SDS-PAGE. Proteins were then transferred onto PVDF membranes (PVDF, Millipore, MA, USA) and blocked with 5% nonfat milk for 1 h at room temperature. After overnight incubation with primary antibodies (N-terminus *β*2AR, NLS2662, Novus Biologicals; C-terminus *β*2AR, sc-570, Santa Cruz Biotechnology; and anti-*β*-actin antibody, sc-58673, Santa Cruz Biotechnology), membranes were incubated with horseradish-peroxidase–conjugated secondary antibodies for another 1 h at room temperature; then immunoreactivity was visualized by enhanced chemiluminescence and X-ray film [15] (GE Healthcare, Piscataway, NJ, USA).

### 2.6. Statistics

All patients' characteristics were expressed as mean ± SD. Comparisons of two groups were analyzed with Student's* t*-test. Correlations were analyzed with Spearman's rank correlation. During the follow-up, hypertension-free survival was defined as the period from OSAS diagnosis till the occurrence of hypertension or the end of follow-up. All cellular experiments were performed for at least three times. *P* < 0.05 was considered statistically significant.

## 3. Results

### 3.1. Characteristics of Enrolled OSAS Patients

**Figure 1:**
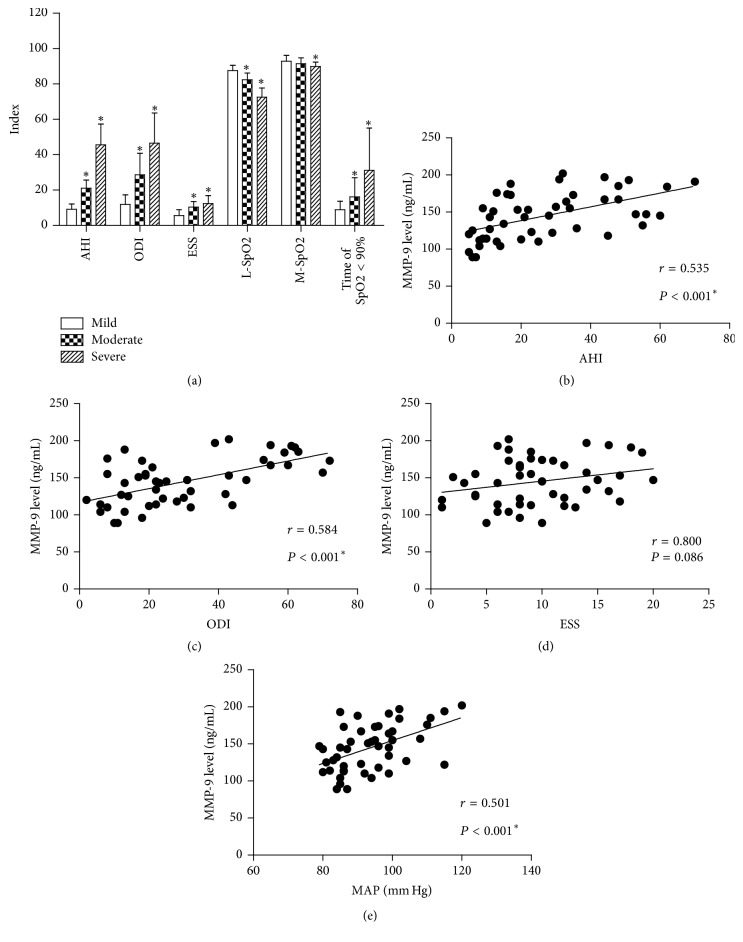
Correlations between MMP-9 and sleep parameters in OSAS patients. (a) General view of polysomnographic parameters according to OSAS severity (Student's* t*-test). The serum MMP-9 level was positively correlated with AHI (b) and ODI (c). Although no significant correlation was observed between MMP-9 and ESS (d), higher serum MMP-9 level (e) was associated with higher MAP according to Spearman correlation test. AHI: Apnea Hypopnea Index; ODI: Oxygen Desaturation Index; ESS: Epworth Sleepiness Scale; SpO_2_: blood oxygen saturation; L-SpO_2_: lowest SpO_2_ during sleep; M-SpO_2_: mean SpO_2_ during sleep; MAP: mean arterial pressure. ^*∗*^*P* < 0.05 compared with mild group.

This retrospective study included 47 cases, aged from 39 to 66 years (median 55.0 years). Among the patients, 16 had mild OSAS, 12 had moderate OSAS, and 19 had severe OSAS. The detailed clinical characteristics and polysomnographic results were presented in Table 1. Patients with moderate/severe OSAS had larger neck and waist circumference than those with mild OSAS. As expected, the AHI, ODI, and ESS indexes were higher in moderate/severe OSAS patients than those with mild OSAS. In addition, the nocturnal desaturation parameters (lowest SpO_2_, mean SpO_2_, and time of SpO_2_ < 90%) also showed significant differences in patients among different disease severities (Figure 1(a)). The BMI, blood pressure, or heart rate showed no statistical difference.

### 3.2. Correlations between Serum MMP-9 Level and Disease Parameters

We next analyzed the correlations between MMP-9 level in the patients' serum with OSAS characteristics by Spearman correlation test. Patients with higher AHI and ODI showed higher MMP-9 levels (Figures 1(b) and 1(c); both *P* < 0.001). However, no statically significant correlation was observed between the MMP-9 level and ESS index (Figure 1(d), *P* = 0.086). Similarly, we calculated the mean arterial pressure (MAP) and identified its positive correlation with MMP-9 expression (Figure 1(e), *P* < 0.001). No significant correlation was observed between MMP-9 and other parameters.

**Figure 2:**
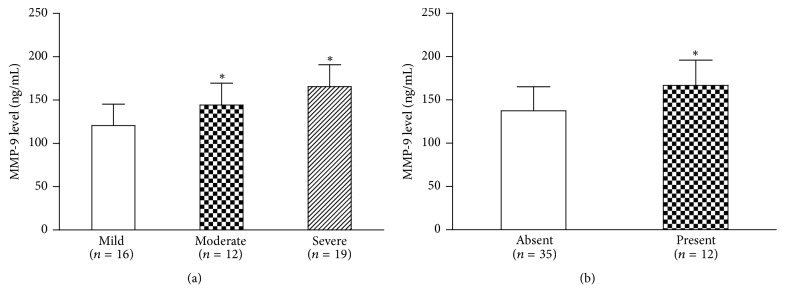
Comparison of serum MMP-9 levels in different OSAS subgroups. (a) Serum level of MMP9 was higher in patients with moderate or severe OSAS (AHI ≥ 15) than that with mild OSAS (5 ≤ AHI < 15). (b) OSAS patients presenting with systematic hypertension (≥140/90 mm Hg) showed higher serum MMP-9 levels (*P* = 0.003). ^*∗*^*P* < 0.05 compared with mild group.

Further statistical analyses were performed by Student's* t*-test to compare the different expression pattern of MMP-9 in subgroups (Table 2). Patients with moderate or severe OSAS showed higher serum MMP-9 levels (144.3 ± 25.2 ng/mL and 165.6 ± 25.2 ng/mL, respectively) than that of patients with mild OSAS (120.6 ± 24.7 ng/mL, Figure 2(a)). Importantly, OSAS patients complicated with hypertension also exhibited elevated MMP-9 levels (Figure 2(b), *P* < 0.001), indicating the crosstalk between MMP-9 and hypertension.

### 3.3. High MMP-9 Level Indicates an Increased Risk of Hypertension and Heart Dysfunction

Taking into consideration our study purpose, we next classified the patients according to the presence of hypertension at the time of diagnosis, as showed in Table 3. None of the parameters showed statistical difference between the two groups, perhaps due to the small case numbers in hypertension group. To determine whether MMP-9 can help predict the occurrence of cardiovascular diseases, we excluded the hypertension patients from our cohort (12/47) and followed up those without any cardiovascular dysfunction (*n* = 35) for up to five years.

We grouped the 35 cases into low-MMP-9 group and high MMP-9 group based on its median level (143 ng/mL). Similarly, patients were classified into various subgroups regarding other parameters to better investigate the risk factors for hypertension (Table 4). By the end of follow-up (March 2017), three patients had left ventricular hypertrophy, and all of them belong to the high MMP-9 group.

**Figure 3:**
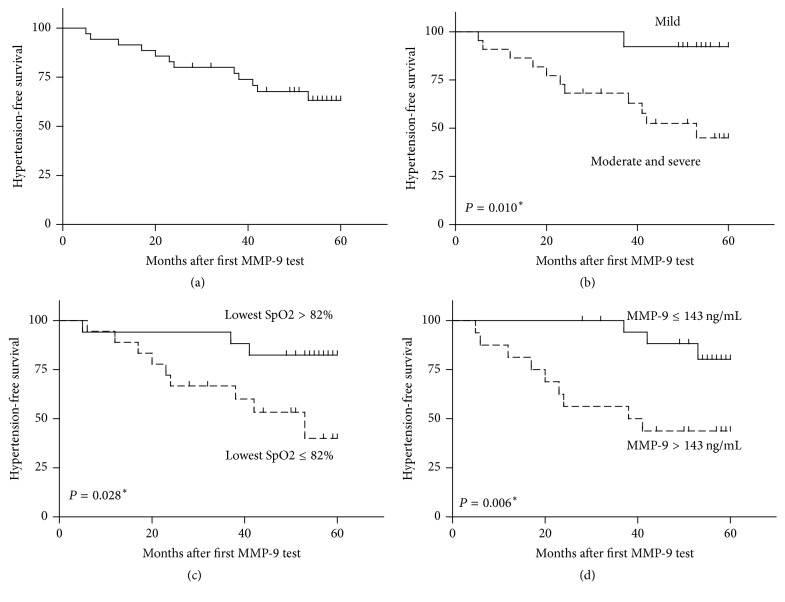
Hypertension-free survival of enrolled OSAS cohort. (a) Till the end of follow-up, 12 cases (12/35, 34.3%) had systematic hypertension. The risk factors for hypertension occurrence include OSAS severity (b), lowest SpO_2_ during sleep (c), and serum MMP-9 level (d). ^*∗*^Significant difference regarding the hypertension-free survival between the two compared groups by Student's* t*-test.

Moreover, 12 cases had systematic hypertension (12/35, 34.2%, Figure 3(a)), emphasizing the high prevalence of hypertension in OSAS patients. By analyzing the hypertension-free survival, we found that OSAS severity (Figure 3(b)), lowest sleeping SpO_2_ (Figure 3(c)), and serum MMP-9 (Figure 3(d)) were all risk factors for hypertension onset.

### 3.4. Hypoxia Upregulates MMP-9 Expression and Enhances *β*2AR Cleavage

**Figure 4:**
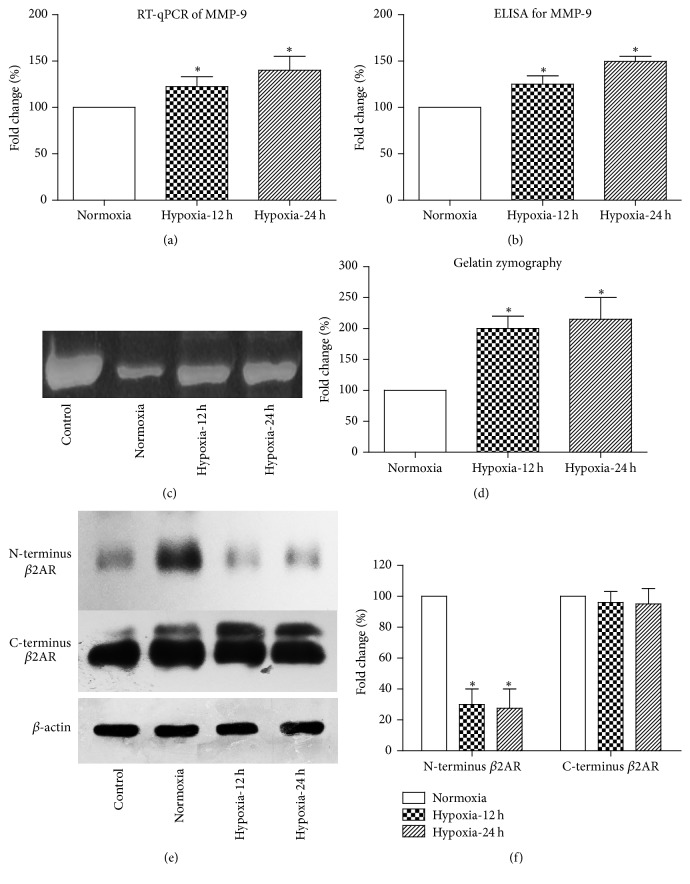
Hypoxia upregulates MMP-9 secretion from vascular endothelial cells and enhances N-terminus cleavage of beta2-adrenergic receptor. The RNA level (a) and protein secretion (b) of MMP-9 were both increased in the rat aorta endothelial cells treated with hypoxia. Cells were cultured in different conditions, and the cell culturing medium was collected for gelatin zymography, which showed an elevated MMP-9 activity in the medium from hypoxia-treated cells (c, d); recombinant MMP-9 protein was used as positive control. The conditional cell culturing medium (from either normoxia or hypoxia conditions) was added to fresh vascular endothelial cells to determine its catalytic role on beta2-adrenergic receptor (*β*2AR); cells in positive control group were supplemented with recombinant MMP-9 protein. By using different antibody epitopes, we found that the N-terminus (extracellular domain) of *β*2AR was cleaved by hypoxia-conditioned medium, while no significant difference was observed on the C-terminus (intracellular domain) of *β*2AR (e, f). The result was consistent with the role of MMP-9 in cleaving extracellular domain of cell surface receptors. All statistical significance was acquired by comparing with normoxia group using Student's* t*-test. ^*∗*^*P* < 0.05 compared with normoxia group.

Beyond this, we cultured rat aortic vascular endothelial cells (VEC) and gave hypoxia condition to mimic the effects of OSAS on human vascular. The RNA levels of MMP-9 were significantly upregulated upon hypoxic treatment (Figure 4(a)). Additionally, the secretion of MMP-9 protein in the cell cultured medium was increased in the hypoxia groups (Figure 4(b)), which was consistent with clinical findings. However, due to the fact that a large proportion of secreted MMP-9 may be pro-MMP-9 without enzymatic activity [16], we further collected the medium supernatant and tested the gelatinolytic activity using gelatin zymography strategy (Figures 4(c) and 4(d)). The zymography results also showed a positive correlation between hypoxia and MMP-9 activity.

Since we determined that both expression and activity of secreted MMP-9 were upregulated by hypoxia, we next explored its effects on vasoconstriction functions. One of the most significant hypertension drug targets is *β*2AR, and there has been a study reported the possible role of *β*2AR as a MMP-9 substrate [17]. Therefore, we collected the cell culture medium from VEC cells, either in normoxia or hypoxia conditions, and added the medium onto fresh primary VEC cells. After culturing with conditioned medium, the surface expression of C-terminus *β*2AR was not changed, while the amount of N-terminus *β*2AR was significantly decreased (Figures 4(e) and 4(f)). The inconsonant results from two antibodies can be explained by the N-terminus (extracellular side) cleavage of *β*2AR by MMP-9 without affecting its C-terminus, implying the involvement of hypoxia-MMP-9-*β*2AR signaling axis in the progression of blood pressure.

## 4. Discussion

Patients with OSAS have a higher prevalence for cardiovascular diseases than healthy individuals, and figuring out the risk factors as well as function mechanisms is significant for OSAS treatment and hypertension prevention. Several mechanisms have been reported to be involved in the development of the cardiovascular diseases in OSAS patients [18]. For example, the endothelial function is dysregulated in OSAS patients (including baseline diameter alteration and high levels of endothelin) and will cause an impaired endothelial-dependent vasodilation [19–21]. Another major hypothesis is correlated with the decreased plasma concentration of nitric oxide (NO) [22] and NO metabolites [23, 24] in OSAS patients. NO, synthesized by endothelial NO synthase (eNOS), is an important protective molecule in vasculature [25]. The eNOS was also reported to be downregulated in OSAS patients [26], resulting in abnormal oxidative stress of the vascular endothelium. The low eNOS level will also cause reduced availability of nitric oxide (NO) and thus impairs endothelial functions by disrupting the balance between matrix metalloproteases and their upstream inhibitors.

Our study showed that a higher serum MMP-9 level in OSAS patients was correlated with hypertension and left ventricular hypertrophy occurrence. Our data is consistent with a previous report that activation of MMP-9 preceded left ventricular remodeling in hypertensive heart failure rats [27]; the researchers also demonstrated that angiotensin-converting enzyme inhibitor (ACEI) can attenuate MMP-9 activity. Therefore, preventively administration of ACEI may be helpful in preventing cardiovascular diseases of OSAS patients, despite the need for further clinical verification. Besides the protein expression, the gene level of MMP-9 also attracted attentions, and it is still under debate whether* MMP-9* polymorphisms affect OSAS occurrence and onset of its complications [28–31].

Importantly, the role of MMP-9 is not restricted to cardiovascular disorders; it is also involved in the progression of microalbuminuria in non-insulin-dependent diabetes mellitus [32]. This is interesting considering many OSAS patients are obese and suffered from diabetes. From this aspect, systematically exploring the clinical significance of serum MMP-9 will be an important breakthrough for elucidating OSAS complication mechanisms.

Since our cellular data revealed that *β*2AR was cleaved by MMP-9, it is possible that the baseline level of *β*2AR in OSAS patients may be dysregulated compared with that in healthy individuals. Beta2AR regulates cardiovascular functions from multiple aspects and previous studies showed that a decreased function of *β*2AR may be involved in hypertension [33]. Polymorphisms of *β*2AR contribute to phenotypic variations in pressor responsiveness and baroreceptor sensitivity and thus are responsible for arterial hypertension [34]. *β*2AR's function of regulating blood pressure depends on its intact protein structure and downstream signaling to induce vascular relaxation [35]. Therefore, the cleavage of *β*2AR by MMP-9 can also partially explain the high prevalence of cardiovascular diseases. Moreover, the high MMP-9 level may exhibit effects on drug resistance of beta-blockers, a class of medications that are commonly used for hypertension treatment.

Our study has several clinical limitations. Firstly, the study group is relatively small because it is not easy to enroll enough untreated OSAS patients without complications. Secondly, the dynamic changes of MMP-9 were not continuously monitored during the follow-up period. Thirdly, OSAS therapy and drug administration during follow-up may also affect the final results. In addition, the up-stream signaling of MMP-9 in hypoxia condition was not investigated in this study; previous data suggested that HIF-1*α* [36], MMP-9 tissue inhibitor [37], and other cytokines [38, 39] may regulate MMP-9 expression. Similarly, since hypoxia and OSAS will cause alterations of multiple biomarkers [5], parallelly analyzing other molecule would be better to predict disease progression.

In all, the current evidence suggests that the serum MMP-9, which may cause cleavage of the extracellular domain of *β*2AR membrane receptor, is closely correlated with the severity of OSAS. An elevated MMP-9 serum level in OSAS patients indicates the unfavorable progression of hypertension and heart dysfunction.

## Figures and Tables

**Table 1:** Characteristics of all OSAS patients.

	Mild OSAS	Moderate OSAS	Severe OSAS
Cases, *n*	16	12	19
Age, year	56.2 ± 6.2	53.2 ± 6.5	51.6 ± 7.9
Male/Female, *n*	9/7	10/2	17/2
BMI, kg/m^2^	27.7 ± 3.5	27.1 ± 2.7	28.4 ± 4.4
Neck circumference, cm	39.1 ± 4.3	42.7 ± 5.0	46.8 ± 4.9^*∗*^
Waist circumference, cm	112.1 ± 8.5	118.2 ± 6.4	121.2 ± 9.8^*∗*^
AHI, events/h	9.2 ± 2.9	21.0 ± 4.6^*∗*^	45.6 ± 11.8^*∗*^
ODI	11.8 ± 5.5	28.6 ± 12.2^*∗*^	46.6 ± 16.9^*∗*^
ESS	5.6 ± 3.2	10.4 ± 3.2^*∗*^	12.4 ± 4.5^*∗*^
Lowest SpO_2_, %	87.5 ± 3.0	82.3 ± 3.8^*∗*^	72.6 ± 5.2^*∗*^
Mean SpO_2_, %	92.8 ± 3.3	91.4 ± 3.3	89.8 ± 2.5^*∗*^
Time of SpO_2_ < 90%, min	8.9 ± 4.8	16.1 ± 10.8^*∗*^	31.1 ± 23.9^*∗*^
Systolic BP, mm Hg	117 ± 14	123 ± 14	129 ± 20
Diastolic BP, mm Hg	76 ± 7	78 ± 7	81 ± 8
Heart rate	69 ± 6	65 ± 6	65 ± 7

Data are mean ± SD; ^*∗*^*P* < 0.05 versus mild OSAS (Student's *t*-test); BMI: body mass index; AHI: apnea/hypopnea index; ODI: oxygen desaturation index; ESS: Epworth sleepiness scale; SpO_2_: blood oxygen saturation; BP: blood pressure.

**Table 2:** Serum MMP-9 level is correlated with the severity of OSAS and hypertension.

	Cases (*n*)	MMP-9 level (ng/mL)	*P* value
OSAS severity			
Mild	16	120.6 ± 24.7	Reference
Moderate	12	144.3 ± 25.2	0.02^*∗*^
Severe	19	165.6 ± 25.2	<0.001^*∗*^
Hypertension			
Absent	35	137.3 ± 27.8	Reference
Present	12	166.7 ± 29.2	<0.001^*∗*^

Data are mean ± SD; ^*∗*^*P* < 0.05 versus reference group (Student's *t*-test).

**Table 3:** Characteristics of OSAS patients with or without hypertension.

	Without hypertension	With hypertension
Cases, *n*	35	12
Age, year	53.4 ± 6.9	54.2 ± 8.0
Male/Female, *n*	26/9	10/2
BMI, kg/m^2^	27.3 ± 3.8	29.3 ± 3.1
Neck Circumference, cm	43.1 ± 5.9	43.4 ± 5.4
Waist circumference, cm	118.7 ± 8.7	118.8 ± 9.2
AHI, events/h	25.5 ± 18.4	31.0 ± 17.0
ODI	27.4 ± 18.1	38.2 ± 22.6
ESS	9.3 ± 4.8	10.5 ± 4.8
Lowest SpO_2_, %	81.4 ± 6.8	76.5 ± 9.4
Mean SpO_2_, %	91.2 ± 3.4	91.2 ± 2.4
Time of SpO_2_ < 90%, min	17.6 ± 18.8	25.9 ± 18.4
Heart rate	65 ± 7	69 ± 6

Data are mean ± SD; All *P* > 0.05, not significant (Student's *t-*test); BMI: body mass index; AHI: apnea/hypopnea index; ODI: oxygen desaturation index; ESS: Epworth sleepiness scale; SpO_2_: blood oxygen saturation.

**Table 4:** Risk factors of hypertension occurrence in OSAS patients.

	Cases	DFS Months	*P* value
Age, year			
≤55	20	51.6 ± 3.2	0.429
>55	15	43.9 ± 5.6	
Gender, *n*			
Male	26	47.6 ± 3.5	0.448
Female	9	49.1 ± 6.2	
BMI, kg/m^2^			
≤23.9	8	46.1 ± 7.0	0.787
>23.9	27	48.9 ± 3.4	
Neck circumference, cm			
≤42.3	19	53.0 ± 3.2	0.204
>42.3	16	42.6 ± 5.2	
Waist circumference, cm			
≤119.0	18	45.0 ± 4.6	0.269
>119.0	17	51.8 ± 3.9	
OSAS severity			
Mild	13	58.2 ± 1.7	0.010^*∗*^
Moderate and severe	22	42.2 ± 4.3	
ODI			
≤22	19	52.8 ± 3.5	0.066
>22	16	42.7 ± 5.0	
ESS			
≤9	19	48.4 ± 4.1	0.869
>9	16	48.2 ± 4.6	
Lowest SpO_2_, %			
≤82	18	42.1 ± 4.6	0.028^*∗*^
>82	17	54.3 ± 3.4	
Mean SpO_2_, %			
≤91	19	45.6 ± 4.2	0.259
>91	16	50.3 ± 4.3	
Time of SpO_2_ < 90%, min			
≤10	18	53.9 ± 3.2	0.074
>10	17	42.1 ± 4.9	
Systolic BP, mm Hg			
≤114	18	50.2 ± 4.4	0.333
>114	17	45.9 ± 4.3	
Diastolic BP, mm Hg			
≤76	18	49.3 ± 3.8	0.660
>76	17	46.4 ± 4.7	
Heart rate			
≤63	18	42.4 ± 5.1	0.123
>63	17	54.5 ± 2.6	
MMP-9 level, ng/mL			
≤143	19	57.0 ± 1.7	0.006^*∗*^
>143	16	37.9 ± 5.4	

All cut-off values are median values, except for BMI (BMI = 23.9 is the cut-off value for normal and obese); ^*∗*^*P* < 0.05 by Student's *t*-test; BMI: body mass index; AHI: apnea/hypopnea index; ODI: oxygen desaturation index; ESS: Epworth sleepiness scale; SpO_2_: blood oxygen saturation; BP: blood pressure.
